# Impact of Digital Educational Interventions to Support Parents Caring for Acutely Ill Children at Home and Factors That Affect Their Use: Protocol for a Systematic Review

**DOI:** 10.2196/27504

**Published:** 2021-06-30

**Authors:** Madison Milne-Ives, Sarah Neill, Natasha Bayes, Mitch Blair, Jane Blewitt, Lucy Bray, Enitan D Carrol, Bernie Carter, Rob Dawson, Paul Dimitri, Monica Lakhanpaul, Damian Roland, Alison Tavare, Edward Meinert, ASK SNIFF Consortium

**Affiliations:** 1 Centre for Health Technology University of Plymouth Plymouth United Kingdom; 2 School of Nursing and Midwifery University of Plymouth Plymouth United Kingdom; 3 Faculty of Health and Society University of Northampton Northampton United Kingdom; 4 School of Sport, Exercise and Health Sciences Loughborough University Loughborough United Kingdom; 5 Faculty of Medicine School of Public Health Imperial College London London United Kingdom; 6 Meningitis Now Gloucestershire United Kingdom; 7 Faculty of Health Social Care and Medicine Edge Hill University Ormskirk United Kingdom; 8 Institute of Infection Veterinary and Ecological Sciences University of Liverpool Liverpool United Kingdom; 9 Meningitis Research Foundation Bristol United Kingdom; 10 Sheffield Children’s NHS Foundation Trust Sheffield United Kingdom; 11 UCL - Great Ormond Street Institute of Child Health University College London London United Kingdom; 12 SAPPHIRE Group Health Sciences University of Leicester Leicester United Kingdom; 13 Paediatric Emergency Medicine Leicester Academic group Children’s Emergency Department University Hospitals of Leicester NHS Trust Leicester United Kingdom; 14 West of England Academic Health Science Network Bristol United Kingdom; 15 Department of Primary Care and Public Health School of Public Health Imperial College London London United Kingdom; 16 See Acknowledgments

**Keywords:** acute disease, telemedicine, child, pediatrics, childhood disease, childhood illness, health education, health literacy, help-seeking behavior, child health, digital intervention, mHealth, primary care, sick child

## Abstract

**Background:**

Urgent and emergency care health services are overburdened, and the use of these services by acutely ill infants and children is increasing. A large proportion of these visits could be sufficiently addressed by other health care professionals. Uncertainty about the severity of a child’s symptoms is one of many factors that play a role in parents’ decisions to take their children to emergency services, demonstrating the need for improved support for health literacy. Digital interventions are a potential tool to improve parents’ knowledge, confidence, and self-efficacy at managing acute childhood illness. However, existing systematic reviews related to this topic need to be updated and expanded to provide a contemporary review of the impact, usability, and limitations of these solutions.

**Objective:**

The purpose of this systematic review protocol is to present the method for an evaluation of the impact, usability, and limitations of different types of digital educational interventions to support parents caring for acutely ill children at home.

**Methods:**

The review will be structured using the Preferred Reporting Items for Systematic Reviews and Meta-Analyses Protocols (PRISMA-P) and Population, Intervention, Comparator, and Outcome (PICO) frameworks. Five databases will be systematically searched for studies published in English during and after 2014: Medline, EMBASE, CINAHL, APA PsycNet, and Web of Science. Two reviewers will independently screen references’ titles and abstracts, select studies for inclusion based on the eligibility criteria, and extract the data into a standardized form. Any disagreements will be discussed and resolved by a third reviewer if necessary. Risk of bias of all studies will be assessed using the Mixed-Methods Appraisal Tool (MMAT), and a descriptive analysis will be used to evaluate the outcomes reported.

**Results:**

The systematic review will commence during 2021.

**Conclusions:**

This systematic review will summarize the impact, usability, and limitations of digital interventions for parents with acutely ill children. It will provide an overview of the field; identify reported impacts on health and behavioral outcomes as well as parental knowledge, satisfaction, and decision making; and identify the factors that affect use to help inform the development of more effective and sustainable interventions.

**International Registered Report Identifier (IRRID):**

PRR1-10.2196/27504

## Introduction

### Background

Uncertainty about the severity of a child’s symptoms has been identified as a key factor prompting parents to seek unscheduled health care and present to emergency departments with acutely ill children or to delay accessing appropriate services [1,2]. Acute illness is typically defined as rapid onset, short-term illness [3,4]. In children, acute illnesses are mainly common, minor conditions like colds, viral rashes, ear infections, or vomiting [5]; however, they can also be severe [3]. Low health literacy has been found to be a factor in parental overestimates of child illness severity, increased urgency for seeking care, and increased use of emergency services [6,7]. There has been an increase in the use of urgent hospital services by children and infants across England in the past decade, including for nonurgent presentations [8-10]. Emergency services are more frequently used by children and young people than adults [8,11]. Estimates of the proportion of nonurgent Accident and Emergency (A&E) attendances range from 15% to 40%, many of which were by young children with minor illness [12]. A study published in 2014 found that approximately 10% of infants (<1 year old) attending A&E had no discernible medical abnormality [13], and a 2017 report of emergency attendance across Yorkshire and Humber determined that there was a 31% rate of nonurgent visits for children (with nonurgent defined for the study as an issue that could have been addressed by a general practitioner) [14]. This behavior is not unique to the United Kingdom; studies around the world have observed high rates of emergency services attendance for nonurgent conditions [15-18], with parental health literary identified as a potential factor in nonurgent attendances [16,17]. The 4-hour A&E target (95% of patients addressed within 4 hours) has not been achieved since 2013 [19], highlighting the current strain on urgent-care hospital resources. A review of factors affecting these behaviors found a range of different reasons, including (among others): parents’ uncertainty and lack of confidence around recognizing problematic symptoms or evaluating their child’s condition; mistrust of, or previous negative experiences with, clinicians; concerns about wasting clinicians’ time; and being perceived negatively by clinicians [20].

This demonstrates the need for better access to primary care services or community-based support for acute pediatric illness and efforts to improve parental health literacy and confidence in determining whether, or which, treatment services are appropriate when a child is ill and how best to manage acute childhood illnesses [1,10,13]. This is particularly relevant in the current context of the COVID-19 pandemic, which has increased the burden on health care services. However, it is important to note that a link has been identified between greater accessibility of primary care services for children and reduced likelihood of visiting emergency services [21]. This suggests that parental educational interventions about recognizing signs and symptoms of acute illness are only one component of the problem and other factors affecting help-seeking behavior for parents with ill children will also need to be addressed in future studies.

### Rationale

Many digital interventions have been developed to provide parents with guidance on how to care for acutely ill children and when it is necessary to seek medical treatment [22-24]. Digital interventions are interventions delivered using medical devices and other digital technologies (as some mobile apps and patient education interventions are not classified as medical devices) [25]. This definition includes a variety of sources such as mobile phones (as apps or text messages), websites, and smart (digitally connected) devices [26]. However, previous systematic reviews have found limited evidence to support the effectiveness of these digital interventions at increasing confidence, reducing anxiety, or improving treatment-seeking decisions [27,28].

The first systematic review to examine this topic was published in 2015 and included educational resources provided in any format: written, verbal, and electronic. It examined a variety of study types and outcome measurements, providing a good overview of the literature [28]. Given the rapid evolution of digital technology [29], the current state of digital interventions to support parents with acutely ill children has likely changed since that systematic review was published. A more recent review (published in 2020) only searched 2 databases and included 3 studies in the final review; it evaluated use and acceptability, accuracy of triage, and use of urgent services [27]. This suggests that it might not provide a sufficiently comprehensive overview of the variety of digital interventions available. Therefore, there is a need for an updated review and evaluation of the state of the literature on digital interventions for parents with acutely ill children to identify what is and is not effective and to inform further innovations.

Conscientious searches of keywords relating to digital intervention, parents, child health, acute disease, and treatment-seeking on PROSPERO [30] failed to find any in-progress systematic reviews on this topic. A new systematic review is needed to identify and evaluate all the published evidence of effectiveness for recently developed digital educational interventions that aim to improve support for parents’ knowledge of acute childhood illness and their confidence and perceived self-efficacy at making the most appropriate care management decisions. An overview of the different types of digital interventions for which there is currently available evidence will help identify promising innovations and areas for improvement in the development and evaluation of these interventions.

The planned systematic review will focus on 4 key research questions to provide this overview. The first 2 questions are based on the research questions of a previous systematic review [28]:

1. How have these digital interventions been developed (eg, what technologies were used, and what steps were taken in their design to ensure accessibility, usability, and acceptability)?
2. What measures are used to evaluate the impact of these digital interventions at achieving their aim?
3. How do current digital interventions impact parents’ knowledge of and experience with managing acute illness at home and use of various health care services for acute childhood illness?
4. What factors influence the usability and user perceptions of these interventions?

## Methods

### Overview

The Preferred Reporting Items for Systematic Reviews and Meta-Analyses Protocols (PRISMA-P) [31] and the Population, Intervention, Comparator, and Outcome (PICO) framework [32] will be used to structure the review. Appropriate Medical Subject Headings (MeSH) will be identified from a preliminary review of the literature. This systematic review will provide an update to a previous systematic review [28]. The first 2 research questions are the same as that previous review, and the third research question was added to include an assessment of usability and sustainability of the interventions, as this is an important component of their success. The systematic review will be composed of a literature search, article selection, data extraction, quality appraisal, data analysis, and data synthesis.

Parents and representatives of groups of parents (eg, Mothers Instinct, Meningitis Now, and Meningitis Research Foundation) were involved in the development and refinement of the review protocol. This involvement is valuable in ensuring that the review represents parents’ perspectives, focuses on issues and questions that are both relevant and true to their experiences, and includes keywords and terms that the researchers might not otherwise have identified.

### Eligibility Criteria

The population, intervention, comparator, outcome, and study (PICOS) type framework (Table 1) was developed in accordance with the review’s research questions.

**Table 1:** Population, intervention, comparator, outcome, and study type (PICOS) framework.

Framework component	Description
Population	Parents and carers of children (aged 0-19 years) will be included. This includes any adult responsible for caring for the child, even if they are not the official guardian (eg, child minders, nursery nurses, teachers, extended family). It will exclude any interventions targeting children or adolescents as the primary user.
Intervention	Any digital intervention (mobile apps, web-based interventions, or smart devices) designed to support parents with acutely ill children by improving knowledge of signs and symptoms of acute childhood illness and decision making about health management and/or treatment-seeking behavior will be included.
Context	Interventions delivered in a variety of settings will be included. This includes both nonclinical settings (eg, homes, schools, and other community settings) and clinical settings (eg, out of hours, primary care, family medicine, general practitioner, ambulatory care, health helplines, and other health care services). Context can refer to both where the recruitment takes place and where the intervention is accessed by the parent. Interventions that recruit or are accessed online will also be included.
Outcomes	The primary objective is to identify the types of digital interventions used to support parents’ health literacy and care of acutely ill children and their effectiveness. Therefore, primary outcomes are expected to include, but are not limited to, health literacy (knowledge and decision making), the confidence in making treatment-seeking decisions and caring for their child, levels of anxiety about the child’s health, actual treatment-seeking behavior, levels and length of engagement with the intervention, and patient-reported experience (including measures of acceptability, usability, or satisfaction). Other outcomes that are reported by studies and deemed relevant will also be included (eg, the ability of the tools to identify a seriously ill child).
Study types	Observational studies (including qualitative studies) and cohort or randomized control trials will be included. Case studies and editorials will not be included. Literature reviews will be included in the search so that their references can be examined to identify any relevant papers not captured by our search terms but will not be included in the final review themselves. Papers describing the development of interventions that are evaluated in one of the studies will also be included.

### Search Strategy

Five databases will be searched to find articles for this review: MEDLINE, CINAHL, Embase, PsycNET, and Web of Knowledge. Key terms relating to digital interventions to support parents with acutely ill children were extracted from an initial review of the literature and used to develop the search terms and search strategy. Search terms will include MeSH terms and keywords relating to digital interventions, children, acute illness, and health information. For this study, acute illness will include any short-term illness, whether minor or severe. Digital interventions will include any digital technologies with the aim of supporting parents or caregivers with children experiencing one or more of these short-term illnesses. An official diagnosis is not required, as the focus of the paper is on how the digital interventions enable parents to respond to children with symptoms of illness. The search terms that will be used in this review are grouped into those 4 themes (see Table 2), and the search string will be created using the following structure: digital interventions (MeSH OR Keywords) AND children (MeSH OR Keywords) AND acute illness (MeSH OR Keywords) AND health education (MeSH OR Keywords). See Multimedia Appendix 1 for a sample search string.

**Table 2:** Search terms.

Category	MeSH	Keywords (in title or abstract)
Digital interventions	Telemedicine OR Mobile Applications OR Internet-based Interventions OR Internet of Things	“mHealth” OR “mobile health” OR “eHealth” OR ((mobile OR phone OR smartphone OR cell) adj3 app*) OR web OR internet OR “online intervention” OR “web-based intervention” OR “digital intervention” OR virtual OR webpage* OR website* OR “smart device*” OR “smart medical devices” OR “smart tech*” OR tool OR resource OR program OR programme
Family	Child OR Infant OR Newborn OR Preschool Child OR Pediatrics OR Family OR Adolescent OR Adolescent Health OR Parents OR Caregivers OR Pregnant Women	pediatric* OR paediatric* OR child OR children OR kid OR kids OR infant* OR newborn* OR neonate* OR bab* OR babies OR toddler* OR schoolchild OR teen* OR adolescent* OR parent* OR carer* OR caregiver* OR “foster parent” OR childminder* OR “child minder*”) OR pregnan*
Acute illness	Acute Disease OR Childhood Disease OR Injury OR Fever OR Cough OR Whooping Cough OR Diarrhea OR Earache OR Vomiting OR Respiratory Tract Infections OR Otitis OR Croup OR Bronchiolitis OR Seizures OR Exanthema OR Mucocutaneous Lymph Node Syndrome OR Conjunctivitis OR Chickenpox OR Epiglottitis OR Tonsillitis OR Common cold OR Influenza, Human OR Pharyngitis OR Meningitis OR Status Epilepticus OR Epilepsy OR Sepsis OR Virus Diseases	(acute OR “short term” OR “short-term” adj2 (illness* OR disease* OR sickness*)) OR (minor adj2 (illness* OR disease* OR sickness*)) OR unwell OR fever* OR febril* OR cough* OR diarrh* OR rash* OR vomit* OR earache* OR bronchiolit* OR (respirator* adj2 infection*) OR otitis OR croup OR seizure* OR rash OR rashes OR exanthem* OR kawasaki* OR conjunctivit* OR “chicken pox” OR chickenpox OR epiglottit* OR tonsillit* OR influenza OR flu OR “sore throat*” OR pharyngit* OR meningit* OR epilepsy OR sepsis OR septicemia OR septicaemia OR epilept* OR headache OR “neck pain”
Health education	Health Education OR Health Literacy OR Help-Seeking Behavior OR Information Seeking Behavior OR Access to Information OR Decision Support Techniques OR Decision Making OR Empowerment OR Prenatal Education OR Health Knowledge, Attitudes, Practice	“Health education” OR “health information” OR “health literacy” OR “information literacy” OR “information resource*” OR “treatment seeking” OR “help seeking” OR educat* OR counsel* OR “consultation behavior*” OR “consultation behavior*” OR (decision adj2 (aid* OR support OR guidance OR help)) OR “parent information” OR “home management” OR empowerment OR confidence OR self-efficacy OR ability OR knowledge OR ?understanding

### Inclusion Criteria

The review will include studies published in English (based in any country) that evaluate digital interventions that aim to improve parents’ health literacy and care for acutely ill children. This will include, but will not be limited to, tools to improve parents’ knowledge of signs and symptoms of acute illness and deterioration, their confidence in assessing illness severity, their perceived self-efficacy in caring for their child, and their treatment-seeking behavior. Digital interventions can include mobile or web-based apps and websites. Studies that examine multicomponent interventions will be included given that there is a digital component of the intervention being evaluated.

The interventions will need to target parents with children (including pregnant women and their families) who have at least one acute illness or to provide education, information, or decision support to prepare parents for the event that a child becomes ill. It is expected that the majority of interventions will target parents with younger children, but the age of 19 years was set as the upper boundary to ensure that no relevant studies are missed in the search. In addition to parents, any caregivers responsible for children (for short or long periods of time) will be included. Studies with any or no comparator will be included.

### Exclusion Criteria

Studies that do not include parents or caregivers responsible for children under the age of 19 years or that target the children (instead of parents or caregivers) as the primary user will be excluded. Depending on the number of eligible references identified in the search, this may be limited to a younger age in the systematic review.

Studies that were published before 2014 will also be excluded for 2 reasons: (1) Digital technology evolves rapidly [29], and this review is concerned with the current state of the field, and (2) this review provides an update and expansion to a previous systematic review conducted in 2014 [28] using 2 of the same research questions and similar search terms. Therefore, studies published before 2014 would likely have been captured in that review.

Studies that merely describe an intervention without evaluating it will be excluded, unless they describe the development of an intervention whose evaluation study is included in the review. Studies that are not published in English will be excluded, as there is no capacity for translation.

### Screening and Article Selection

The citation management software EndNote X9 will be used to store the references and automatically remove any duplicates. References will be uploaded to a meta-analysis software to facilitate initial screening (based on inclusion and exclusion criteria key words), data extraction, and analysis. Two independent reviewers will then screen the remaining titles and abstracts and then conduct a full-text review to determine final eligibility for inclusion. Any disagreements about eligibility will be discussed by the 2 reviewers, and if no consensus can be reached, eligibility will be decided by a third reviewer. The details of the screening and selection process will be recorded using a PRISMA flow diagram to ensure study reproducibility.

The references of any relevant reviews found in the initial search will also be screened to identify any studies that may have been missed by the search. Once the final set of included studies has been determined, their references will be searched for published papers describing the development of those interventions. These linked papers will also be included in the final review.

### Data Extraction

Two reviewers will independently examine the full texts of the included articles to extract outcomes into a predetermined form (see Textbox 1). Where the index paper does not include sufficient information about intervention development, linked (cited) publications will be used to provide the required data. As there are expected to be a variety of outcomes reported and not all are likely to have been anticipated, relevant outcomes reported by the studies that are not included in this table will be included in the final review. As with the screening, disagreements will first be discussed and then settled by a third reviewer if necessary.

**Article information and data to be extracted.**

General study information

- Year of publication
- Country of study
- Sample demographics (including, but not limited to, any of the following that are reported: age, gender, target population, parental experience, socioeconomic status, health literacy, locality, health conditions)
- Initial sample size
- Analyzed sample size
- Length of follow-up

Intervention

- Digital platform
- Cost
- Development methods addressing accessibility/implementation
- Aim of intervention
- Intended time and place of use (eg, before seeking help, after seeking help)
- Training or guidance needed to use (if any)
- Specified age of children (if any)
- Specified type of acute illness (if any)
- Theory or logic model the intervention is based on (if any)
- Patient and public involvement in development (if any)

Evaluation

- Outcomes measured
- Health literacy (knowledge of illness and decision making); as there are a variety of tools used to measure health literacy [33], both the tool used and the finding will be extracted
- Skills to manage child illness
- Parental treatment-seeking behavior
- Parental characteristics (eg, uncertainty, anxiety, knowledge, confidence, reassurance, perceived self-efficacy)
- Acceptability
- Usability of platform
- Accessibility
- User experience (participant perceptions or feedback)
- Sustainability of use
- Other key performance indicators reported (eg, ability of tools to identify a seriously ill child)
- Limitations identified

### Quality Appraisal and Risk of Bias Assessment

The quality and risk of bias of the included studies will be independently assessed by 2 reviewers, with disagreements discussed and resolved by a third reviewer if necessary. They will be measured using the Mixed-Methods Appraisal Tool (MMAT) [34]. Although this is a newer tool that has not been as comprehensively validated as other quality assessments, it was chosen because it will enable all of the included studies to be consistently assessed using the same criteria. The quality of all included randomized controlled trials and their overall performance for each bias will be summarized in figures.

### Data Analysis and Synthesis

A meta-analysis is not expected to be feasible, due to the anticipated variety of study designs, measures, and reported outcomes. A descriptive analysis will be used to summarize the extracted data. The studies will also be analyzed separately depending on the age of the children. Where possible, they will be divided into 4 groups (0-4, 5-9, 10-14, 15-19 years) to align with the division used by Public Health England and the World Health Organization [35,36] and to allow comparison with national statistics. As there is a lack of standardized age bands for childhood, it is possible that some of the studies will target parents with children of ages that do not fit into a particular age group. If this occurs, it will be noted in the review and analyzed with the group(s) with which it is best aligned. The age-divided analysis will be conducted in addition to a general analysis to explore the possibility of age-related differences in interventions and their outcomes.

### Patient and Public Involvement (PPI)

Our approach reflects best practice in health research [37]. Parents are central to the review, not only as expert team members but also in the search for information concerning how parents have been involved in the development, delivery, and evaluation of the interventions identified in our review.

Parents and representatives of groups of parents will continue to be involved in the review process. We do not expect patient and public involvement (PPI) experts to review individual papers as this is not their area of expertise. However, 2 representatives from meningitis awareness charities (JB, Meningitis Now, and RD, Meningitis Research Foundation) were involved in the revision of this protocol. We will ask parents and PPI representatives to review our findings from the review of included papers to ensure that any factors that may have affected parents’ participation in projects are identified and the interpretation of the findings are grounded in the reality of life as a parent. In this way, we intend to ensure that the review is not biased towards an academic or clinical lens.

## Results

The systematic review is expected to start in May 2021 and be completed by December 2021. However, given the current public health emergency, a firm timeline cannot be guaranteed.

## Discussion

A systematic review of the literature about digital interventions to support parental decision-making for, and care of, acutely ill children will contribute to a better understanding of how these interventions can best support parents. Based on the data about impact, usability, and limitations that will be extracted from the studies, this section will explore what conclusions can be drawn, the limitations of the systematic review, and key areas for future research.

A better understanding of the current strengths and areas for improvement of these digital interventions has the potential to promote timely use of primary care services according to the severity of illness of the child. Given the lack of substantial evidence supporting the effectiveness of such interventions identified by previous reviews, the conclusions drawn from this review will help inform the development of improved digital interventions for parents. This will be particularly important for designing interventions to improve access for hard-to-reach populations and others who are vulnerable to digital exclusion.

## Appendix

Multimedia Appendix 1 Sample search.

## Abbreviations

A&E: Accident and Emergency
MeSH: Medical Subject Headings
MMAT: Mixed-Methods Appraisal Tool
PICO: Population, Intervention, Comparator, and Outcome
PPI: patient and public involvement
PRISMA-P: Preferred Reporting Items for Systematic Reviews and Meta-Analyses Protocols
