# Supplementary material for: Impact of Digital Educational Interventions to Support Parents Caring for Acutely Ill Children at Home and Factors That Affect Their Use: Protocol for a Systematic Review
Source: JMIR Res Protoc. 2021 Jun 30;10(6):e27504. doi: 10.2196/27504 (PMC8280832; doi:10.2196/27504)
Supplement: Multimedia Appendix 1 [file resprot_v10i6e27504_app1.docx]

Appendix 1. Sample Search

| Database | Search String | Articles |
| --- | --- | --- |
| PubMed | ((Telemedicine OR Mobile Applications OR Internet-based Interventions[MeSH Terms]) OR (“mHealth”[Title/Abstract] OR “mobile health”[Title/Abstract] OR “eHealth”[Title/Abstract] OR ((mobile OR phone OR smartphone OR cell) adj3 (app OR apps OR application*))[Title/Abstract] OR web[Title/Abstract] OR internet[Title/Abstract] OR online intervention[Title/Abstract] OR web-based intervention[Title/Abstract] OR digital intervention[Title/Abstract] OR virtual[Title/Abstract] OR webpage*[Title/Abstract] OR website*[Title/Abstract])) AND  ((Child OR Infant OR Newborn OR Preschool Child OR Pediatrics OR Family[MeSH Terms]) OR (Pediatric*[Title/Abstract] OR paediatric*[Title/Abstract] OR child[Title/Abstract] OR children[Title/Abstract] OR kid[Title/Abstract] OR kids[Title/Abstract] OR infant*[Title/Abstract] OR newborn*[Title/Abstract] OR baby[Title/Abstract] OR babies[Title/Abstract] OR toddler[Title/Abstract])) **AND** ((Acute Disease OR Childhood Injury OR Childhood Disease OR Injury OR Fever OR Cough OR Whooping Cough OR Diarrhea OR Earache OR Vomiting OR Respiratory Tract Infections OR Otitis OR Croup OR Bronchiolitis OR Seizures OR Exanthema OR Mucocutaneous Lymph Node Syndrome OR Conjunctivitis OR Chickenpox OR Epiglottitis OR Tonsillitis OR Common cold OR Influenza, Human OR Pharyngitis OR Meningitis OR Status Epilepticus OR Epilepsy OR Sepsis[MeSH Terms]) OR ((acute adj2 (illness*[Title/Abstract] OR disease*))[Title/Abstract] OR (minor adj2 (illness*[Title/Abstract] OR disease*[Title/Abstract] OR injury))[Title/Abstract] OR fever*[Title/Abstract] OR febril*[Title/Abstract] OR cough*[Title/Abstract] OR diarrh*[Title/Abstract] OR rash*[Title/Abstract] OR vomit*[Title/Abstract] OR earache*[Title/Abstract] OR bronchiolit*[Title/Abstract] OR (respirator* adj2 infection*)[Title/Abstract] OR otitis[Title/Abstract] OR croup[Title/Abstract] OR seizure*[Title/Abstract] OR rash[Title/Abstract] OR rashes[Title/Abstract] OR exanthem*[Title/Abstract] OR kawasaki*[Title/Abstract] OR conjuctivit*[Title/Abstract] OR "chicken pox"[Title/Abstract] OR chickenpox[Title/Abstract] OR epiglottit*[Title/Abstract] OR tonsillit*[Title/Abstract] OR influenza[Title/Abstract] OR flu[Title/Abstract] OR "sore throat*"[Title/Abstract] OR pharyngit*[Title/Abstract] OR meningit*[Title/Abstract] OR epilepsy[Title/Abstract] OR sepsis[Title/Abstract] OR epilept*[Title/Abstract]))) **AND** ((Health Education OR Health Literacy OR Help-Seeking Behavior OR Information Seeking Behavior OR Access to Information OR Decision Support Techniques OR Decision Making[MeSH Terms]) OR ("Health education"[Title/Abstract] OR “health information”[Title/Abstract] OR “health literacy”[Title/Abstract] OR "information literacy"[Title/Abstract] OR "information resource*"[Title/Abstract] OR “treatment seeking”[Title/Abstract] OR “help seeking”[Title/Abstract] OR educat*[Title/Abstract] OR counsel*[Title/Abstract] OR "consultation behavior*"[Title/Abstract] OR "consultation behaviour*"[Title/Abstract] OR (decision adj2 (aid[Title/Abstract] OR aids[Title/Abstract] OR support[Title/Abstract] OR guidance[Title/Abstract] OR help))[Title/Abstract])) | 1,313  950 (published after 2014) |
